# Development and evaluation of a culturally appropriate hypertension education (CAHE) training program for health care providers

**DOI:** 10.1371/journal.pone.0178468

**Published:** 2017-06-08

**Authors:** Jennita G. Meinema, Joke A. Haafkens, Debbie A. D. C. Jaarsma, Henk C. P. M. van Weert, Nynke van Dijk

**Affiliations:** 1 Department of General Practice/ Family Medicine, Academic Medical Center-University of Amsterdam, Amsterdam, the Netherlands; 2 Amsterdam Institute for Advanced labour Studies, University of Amsterdam, Amsterdam, the Netherlands; 3 Center for Education Development and Research in Health Professions, University Medical Center of Groningen, University of Groningen, Groningen, the Netherlands; Universite de Bretagne Occidentale, FRANCE

## Abstract

**Background:**

In Western countries, hypertension and hypertension-related complication are more common in ethnic minority groups of African descent than in indigenous populations. Addressing ethnic minority patients’ perceptions of hypertension and its treatment through the use of cultural appropriate hypertension education (CAHE) increases adherence to medication and lifestyle recommendations. Given these effects, it seems warranted to develop a training program on how to deliver this type of patient education for Primary Care Nurse Practitioners (PCNPs).

**Objective:**

Development and evaluation of a training program for PCNPs aimed at providing culturally appropriate hypertension patient education.

**Design:**

Prospective cohort study evaluating attitude and intended behavioral changes.

**Participants:**

Both experienced PCNPs and PCNPs in training participated in this study.

**Main measures:**

The effects of the CAHE-training were measured by 3 different questionnaires on 1) the satisfaction with the training program, 2) the attitude towards culturally appropriate care, and 3) the commitment to change.

**Results:**

The CAHE-training program consists of 10 different components divided over two 4-hour sessions and was taught to 87 participating PCNPs. The program utilizes constructivist-learning principles and educational evidence on adult learning. The content of the program is based on the knowledge obtained from our previous studies on culturally appropriate care. The mean satisfaction-score was 7.5 (1–10 scale), with the role-play exercise with patient-actors scoring highest (8.2). We observed non-significant but positive changes in attitude. PCNPs who reported on the implementation of their intended behavior change showed significant attitude changes after three months.

**Conclusion:**

We demonstrated that our evidence based training program for PCNPs resulted in a positive learning experience with adequate intended behavioral changes in practice. Unfortunately, response rates were too low to demonstrate persistent changes in attitude.

## Introduction

Hypertension is a major risk factor for cardiovascular disease. [1] In Western countries, hypertension is more common among ethnic minority groups of African descent than among the indigenous population [2–4]. Among Dutch hypertensive patients, blood pressure control rates are also significantly lower among African Surinamese and Ghanaians than among native patients [5, 6]. In the Netherlands, the primary care treatment of patients with hypertension includes regular meetings with Primary Care Nurse Practitioners (PCNPs), who perform protocolized check-ups, monitor medication prescriptions, and provide lifestyle recommendations. The treatment outcomes are most optimal when patients comply with medication prescriptions and lifestyle recommendations [7, 8]. Therefore PCNPs are commonly trained in methods to facilitate adherence (like motivational interviewing).

Adherence, however, is lower among ethnic minority groups than among the indigenous population [9]. In North America and Europe several interventions were developed to address specific risk factors of hypertension and treatment adherence in ethnic minority groups [10, 11]. These interventions relate explicitly to the cultural perspective of patients. We previously developed an educational intervention that delivered cultural specific hypertension education to patients (CAHE intervention). The CAHE intervention typically combines the principles of Motivational Interviewing (MI) [12] with concepts of Arthur Kleinman’s theory of explanatory models, which proposes that patient perspectives of disease and treatment are influenced by their socio-cultural environment and may differ substantially from the medical perspectives of their healthcare providers [13]. The intervention was aimed at promoting adherence to lifestyle and medication recommendations and blood pressure control in hypertensive patients of West African descent [14]. In addition to written patient education materials, it includes a series of essential questions that patient educators (PCNPs) can ask in order to help patients understand their health beliefs and their health condition, and to adapt their health behaviors so as to improve their blood pressure. The CAHE intervention was piloted in a randomized controlled study. This study demonstrated CAHE led to positive outcomes among African-Surinamese and Ghanaian patients such as a better understanding of hypertension, greater concern about the associated risks, and more awareness that hypertension can be controlled but not cured [15]. Most importantly, African-Surinamese and Ghanaian patients who received CAHE showed a reduced diastolic blood pressure (DBP) and generated better adherence to lifestyle recommendations than those did not [14, 16]. Given these results, the wider use of CAHE in general practice seemed warranted.

To translate the results of our pilot-intervention into medical practice, we created an interactive, comprehensive, and reproducible training program on how to use CAHE for PCNPs. In this article, we discuss the development of this training program. In addition, we evaluate the effectiveness of the training by assessing its effects on attitudes of PCNPs with respect to culturally appropriate education and on the implementation of intended behavior changes in practice.

## Methods

To develop the CAHE-training program, we followed the curriculum model of Prideaux [17]. This model encompasses five major elements and their interactions: context, content, statements of intent (goals and objectives), teaching and learning strategies, and assessment strategies. We describe the development of the CAHE-training in concordance with these five elements.

### Context

In the Netherlands, 13 Universities of Applied Sciences offer professional degree programs for PCNPs. The programs only accept students who are experienced health care providers (doctor's assistants or nurses). The curriculum takes 1–2 years depending on the student’s previous experience and education [18] and it covers a limited number of specific care domains including type 2 diabetes, asthma/COPD, cardiovascular diseases, and geriatrics [18]. The training program developed in this project was incorporated in the module cardiovascular diseases which is offered at the end of the 2-year educational program. Both experienced PCNPs and PCNPs in training were allowed to participate in the CAHE-training program. Experienced PCNPs paid a tuition fee (150 euro) and received accreditation or educational credits on completion. We informed all participants of the CAHE program about the study, only those who gave written informed content were included. All the PCNPs in training were explicitly informed in writing that their course evaluation was part of this study and that participation in the evaluation was voluntary. Submission of the questionnaires was considered as implied consent. We kept all test scores confidential and removed identifiers directly after the test. The coding list was available to the first author (JM) only.’ Since institutional review boards in the Netherlands only consider studies that included patients, we did not submit the study plan for review. The Medical Ethics Committee of the University of Amsterdam exempted the study from formal ethical approval.

### Content

#### Scientific foundation of the CAHE-training

The content of the CAHE-training is based on knowledge obtained from previous projects. The first project explored and compared perceptions on hypertension among native Dutch and first-generation Ghanaian and African-Surinamese hypertensive patients [19]. This study showed that Ghanaian and Surinamese patients attributed hypertension to different causes than health-care providers, for example to migration-related factors such as changes in diet, climate or stress that is associated with living in a new country.

In the second project, we developed a multi-component provider intervention to facilitate culturally appropriate hypertension patient-education (CAHE-intervention), combining Motivational Interviewing (MI) [12] with the principles of Arthur Kleinmans’ model [13]. PCNPs who had received the intervention considered it more important to take a patient's cultural background into account when delivering care [20]. The intervention also effectively lowered barriers to deliver CAHE-care to hypertensive patients [21].

The third project described the CAHE-intervention and tested the effectiveness among migrant patients [14]. In addition to standard care, the intervention group was offered three CAHE sessions led by a PCNP. This intervention led to significant improvements in diastolic blood pressure and adherence to lifestyle recommendations.

The fourth project identified the patient-related determinants of adherence to lifestyle and medication recommendations among Surinamese and Ghanaian patients and how CAHE-intervention influenced those determinants [15]. Medication adherence was related to patients’ medication self-efficacy, the concerns about the medication use, and patients’ perceptions on hypertension. It demonstrated that the CAHE-intervention improved patients’ understanding of hypertension and lead to more concerns about the associated risks and a greater awareness of the chronic nature of hypertension.

#### Content of the CAHE-training

Based on the results of the studies described above, an evidence-based educational program for PCNPs was constructed by the educational research team of the Department of General Practice at the Academic Medical Centre in Amsterdam. The design of the CAHE-training was based on constructivist learning theories, which emphasize that “learning takes place through interactions with other students, teachers, and the world-at-large”. These theories state that knowledge and skills are acquired best by associating new information with existing knowledge [22]. Since PCNPs are trained and experienced in applying Motivational Interviewing, which is also one of the building blocks of the CAHE-training, they should be able to adopt the training material quickly.

Two pilots of the CAHE-training were conducted, one with experienced PCNPs (n = 17, pilot 1) and the other with PCNPs in training (n = 21, pilot 2). Minor changes were made based on the results of these pilot studies and suggestions for improvement made by the participants. After the pilot studies, we reduced the duration of the theoretical part to allow more time for role-play exercises and the number of teachers was limited to two. Furthermore, the introduction (learning objectives and theory/background information) was provided digitally to PCNPs before the start of the training, instead of at the start of the first meeting.

The final CAHE-training program consisted of two four hour sessions divided over ten different components (Table 1). During the first session, participants were taught by classroom teaching, which included interactive lectures and video materials. During the second session, PCNPs were divided in groups of 3–4 person, to stimulate interaction so as to ensure that the educational experience (of exercising) was effective [23]. The methods used to achieve the different components and to test the learning results of students are shown in Table 1.

**Table 1:** Description of the CAHE-training program and teaching, learning and assessment methods of the CAHE-training program.

**Duration**	**Day 1**	**Teaching methods**	**Learning strategies**	**Assessment**
30 min	1. Introduction (pre-test, needs assessment and purpose and method of the program)	Lecture		
30 min	2. State of the art: treatment of hypertension in bird flight	Interactive lecture	Repetition	
20 min	3. Difficulties in hypertension care for immigrant patients	Classroom discussion	Enabling	
55 min	4. Why CAHE?	Lecture		
20 min	5. How CAHE?	Interactive lecture		
45 min	6. Examples of CAHE (video)	Interactive lecture with film and audio materials	Enabling	
15 min	Explanation of the homework assignment		Facilitating: activate prior knowledge	Exercise/ self-study assignment
**Duration**	**Day 2**	**Teaching methods**	**Learning strategies**	**Assessment**
15 min	7. Introduction and explanation exercise CAHE	Lecture		
3 rounds of 60 min	8. Discussion of the homework assignment 9. Exercising CAHE with patient-actor 10. Discussion of the exercise with the patient-actor	Roleplay (9)/ workgroup (8,10)	Reinforcing: applying CAHE-care	Self-study assignment, Role-play (Formative feedback)
30 min	Evaluation and reflection	Interactive lecture		

The participants are introduced to the program subject by a homework assignment. The aim of this assignment is to activate prior knowledge and to formulate personal learning goals (facilitating). After an overview of the state of the art approach in hypertension care, the PCNPs discuss the challenges they experience when providing care to immigrant patients (enabling). Next, we introduce CAHE-care and explain why it is important. Finally, the participants apply CAHE-care to a patient-actor and discuss their experience in work groups (reinforcing).

#### Statements of intent (goals and objectives)

At the end of the program, PCNPs were expected to be capable of providing CAHE in primary care practice. The specific learning objectives for the PCNPs are distributed over four objective themes as specified in Table 2. Additionally, the pyramid of Miller is used to specify at which competence level (knows, knows how, shows how or does) the objectives of the program should be achieved [24].

**Table 2:** Learning objectives and competence level (based on Miller [24]).

Objective theme	Specific objective	Miller
CAHE and attention for CAHE in own practice	The student can explain what culturally appropriate education is.	Knows
	The student can indicate the importance of CAHE-care.	Knows
	The student can identify which patients need CAHE-care.	Knows how
Differences between personal frame of reference of health and disease, and the frame of an immigrant patient	The student can express his/her own medical perspectives, (cultural) values, norms and principles.	Knows
	The student can explain his/her own medical perspectives, and his/her attitude with respect to migrant patients.	Knows how
	The student knows the difference between the ‘perceptions / medical reference framework’ of the migrant patients and its own.	Knows how
	The student acknowledges that cultural factors influence the perception of illness and health of migrant patients.	Knows how
Tools and stadia of the education to apply CAHE-care	The student can explain the two different tools to apply culturally appropriate education and why these tools have been developed.	Knows how
	The student can identify and explain the purpose of the three stadia of culturally appropriate education.	Knows how
	The student recognizes the barriers (of each stadium) during culturally appropriate education and how to deal with these barriers.	Knows how
Skills to apply CAHE-care	The student possesses skills to elicit and integrate the perceptions, interpretations, and conceptions of migrant patients with regard to their health, illness, and social environment (on the basis of MI-techniques).	Shows how
	The student is able to discuss how the cultural background, migrant history, illegality, and discrimination affect the situation and healthcare needs of migrant patients.	Shows how

#### Teaching and learning strategies

To ensure consistency in the way the training was taught, six teachers (general practitioners, psychologists, and educationalists) were trained. The training focused on the principles and theories of culturally appropriate care, active learning (constructivism), and on the rationale behind the design of the CAHE-training. Teachers were provided with all training materials (visual aids, hand-out materials, homework assignments, role-play materials, speaker notes, and a comprehensive handbook) for each session (available on request from first author). The handbook contained details of the clinical evidence behind the training, training organization, learning objectives, scripts for each component of the training, tools, simulation materials, and links to supplemental materials. The CAHE-training utilizes a needs assessment used to adapt the lectures, multiple methods of teaching, and various learning strategies (facilitating, enabling, and reinforcing) (Table 1) [25].

#### Assessment strategies

During the program, role-play exercises and self-study assignments were used as a formative assessment tool (Table 1). These tools allowed PCNPs to practice in applying their attained knowledge and receive feedback on their performance before applying CAHE-care in practice. Based on the principles outlined by the four previous studies [14, 15, 19, 20], trainees received specific formative feedback about their attitude towards the patient-actor, application of tools, and how they considered the cultural aspects of the patient.

### Evaluation of the program: Questionnaires

To evaluate the effects of the CAHE-training on PCNPs attitudes towards cultural specific care and intended behavior change, the participants were requested to fill out multiple questionnaires. The questionnaires included items on 1) satisfaction with the training program (immediately after the training program), 2) attitude (before and three months after the training program) and 3) commitment to change (immediately after and three months after the training program).

1. The items to assess satisfaction with the training (N = 32) covered the following aspects: overall quality, content, objectives, teaching, learning and assessment strategies, and perceived learning outcomes. Five were open questions. The 27 closed questions could be answered on a 5-point Likert scale.
2. The items to measure participants’ attitude (N = 11) were selected from three other questionnaires that were used to measure healthcare providers’ towards culturally competent care [26]. The main themes covered by the items were: ‘how important do you consider the patients’ culture when providing care’ (N = 5 items), and ‘how often do you consider a patient's cultural background while providing care’ (N = 6 items). The questions could be answered on 5-point Likert scale.
3. Reflection is important in effecting change in practice [27]. We evaluated “commitment to change” (CtC) by asking the participants to define, in writing, three goals with respect to intended behavioral changes related to culturally appropriate care for minority patients. Three months after completion of the program, the PCNPs were sent a copy of their original commitment-to-change forms with a request to indicate to what extent they had implemented their commitment-statements in practice on a 7-point Likert scale. Based on their answers a composite score for implemented behavioral changes was computed (range: 1–7). PCNPs with scores ≤4 were categorized as ‘did not implement’ the intended behavior change and those with scores 5 or higher as ‘implemented’ the intended behavior change. Additionally, the PCNPs were asked to clarify their implementation score. The period of three months was chosen to ensure that PCNPs treated sufficient numbers of migrant patients to be able to implement the intended changes into their practices.

### Analysis

We calculated the mean scores of the satisfaction and attitude items using IBM SPSS version 22.0. The mean attitude scores before and after three months were compared using a chi-square test for categorical data and an independent paired t-test for normally distributed continuous data. All CtC statements listed on the forms were reviewed and categorized by two researchers (JGM and NvD). Where applicable, statements were assigned to one of the overarching objectives (Table 2) or otherwise under ‘other objectives’.

## Results

The evaluated CAHE-training program was offered on nine occasions at four different locations throughout the Netherlands from 2012–2015. In total, 102 PCNPs finished the training, 87 of who completed the satisfaction questionnaire directly after completing CAHE-training. These 87 PCNPs were included in the evaluation of the training. The demographic characteristics of these participants are shown in Table 3.

**Table 3:** Characteristics of the total group PCNPs.

Demographic characteristics	Total group PCNPs (87)
Gender (female), n (%)	96.3
Age, mean (sd)	44.6 (10.1)
Origin (immigrant), n (%)	15 (17.2)
Practice experience in years, mean (sd)	2.9 (4.1)

### Satisfaction

The mean satisfaction score with the program was 7.5 (sd = 1.2) (1–10 scale). The role-play with a patient-actor scored the highest at 8.2 (sd = 1.1) (1–10 scale). Participants appreciated the multitude of different learning methods and the novelty of the culturally appropriate approach (as reported on the open questions of the satisfaction questionnaire).

### Attitude

Of the 87 participants who completed the attitude before following the program, 31 (36.6%) completed the attitude questionnaires three months after the program. We compared the scores of the PCNPs before the CAHE-training and after three months and observed only minor, non-significant differences (Table 4, column 2 and 3). At the start of the study, migrant PCNPs seem more aware of cultural differences while providing care for minority patients (Table 4, column 4 and 5). The mean attitude score on 2 out of 11 questions are significantly higher for immigrant PCNPs than for indigenous PCNPs (p < 0.03 and p < 0.05).

**Table 4:** The mean attitude scores of the total group, immigrant vs indigenous PCNPs, and the mean difference scores for PCNPs who implemented the CtC statements and those who did not.

Attitude questionnaire (1 = not important to 5 = very important).	Mean (sd) score at the start of the program (n = 86)	Mean (sd) score after three months (n = 31)	Mean (sd) score for immigrant PCNPs at the start of the program (n = 15)	Mean (sd) score for indigenous PCNPs at the start of the program (n = 71)	Mean difference (sd) for PCNPs who implemented the CtC statements in their practice (n = 19)	Mean difference (sd) for PCNPs who did not implemented the CtC statements in their practice (n = 8)
How important do you consider the patient’s culture to be when providing care:						
• to those from cultures different from your own	4.3 (0.69)	4.3 (0.68)	4.6^1^ (0.51)	4.2^1^ (0.70)	-0.18	-0.11
• to those with health beliefs or practices at odds with western medicine	4.0 (0.79)	4.1 (0.83)	3.9 (0.83)	4.1 (0.79)	0.23	0.28
• to those who distrust the Dutch health care system	4.0 (0.93)	4.1 (0.96)	4.1 (1.13)	4.0 (0.89)	0.14	0.69^2^
• to those who are members of ethnic minorities	3.8 (0.80)	3.9 (0.72)	3.9 (0.88)	3.8 (0.78)	-0.18	0.02
• to those whose religious beliefs affect treatment	3.9 (0.94)	4.0 (0.91)	4.3 (0.82)	3.9 (0.95)	-0.13	0.00
How often do you consider a patient's cultural background while:						
• determining how a patient wants to be addressed and interacted with	3.2 (0.78)	3.2 (0.67)	3.4 (0.63)	3.1 (0.80)	0.01	0.19
• performing an anamnesis	3.3 (0.72)	3.4 (0.61)	3.4 (0.74)	3.3 (0.72)	0.07	0.22
• eliciting patients' understanding of illness	3.13 (0.78)	3.10 (0.54)	3.33 (0.72)	3.08 (0.79)	-0.06	0.51^2^
• eliciting patients' perceptions regarding prescribed medication	3.19 (0.81)	3.17 (0.59)	3.20 (0.78)	3.18 (0.82)	0.32	0.51^2^
• identifying patients' customs that might affect adherence to clinical care	3.17 (0.72)	3.32 (0.54)	3.33 (0.49)	3.14 (0.76)	0.01	0.35
• assessing the influence of family or community members on adherence to clinical care	2.90^1^ (0.81)	3.13 (0.67)	3.27^1^ (0.59)^1^	2.82^1^ (0.83)^1^	0.23	0.32

^1^ Immigrant PCNPs mean score is at the start of the program significant higher on these questions (p = 0.03 and p = 0.05)

^2^ PCNPs who reported implementation of their CtC statements score significantly higher on these questions (p = 0.04, p = 0.03 and p = 0.04)

### Commitment to change

Out of the 87 participants, 30 (34.5%) completed the Commitment to Change questionnaire after three months. The number of CtC statements varied per PCNP from 1 to 3. The majority (56.8%) of the statements were linked to the major topic/objective (skills to apply CAHE-care) of the program. (Table 5). The mean implementation score was 5.1 (out of 7), and 70.4% of the statements were said to be implemented (score of 5.0 or higher). More than 70% of the statements were associated with general MI concepts (not culture specific). Some illustrative examples of non culture specific commitment to change statements are: “Give the patients more time to respond and grasp the information he just received”, “Summarize regularly and ask the patients whether he understands the explanation”, and “First contact, then contract”. Some commitment to change statements were culture specific, for example: “More hesitant in providing solutions. Chat about culture and make the patient think”, “More considerate on different views from other cultures concerning the causes and symptoms”.

**Table 5:** CtC statements associated with topic/objectives of the program.

Objective theme	Intended changes (126)	Cited (%)	Cultural-specific (%)	MI-specific (%)
1. CAHE-care and attention for this subject in own practice	9	8.1	100	0
2. Differences between personal frame of references of health and disease, and the frame of an immigrant patient	17	15.3	33.3	66.7
3. Tools and stadia of the education to apply CAHE-care	9	8.1	22.2	77.8
4. Skills to apply CAHE-care	63	56.8	20.6	79.4
5. Other objectives	13	11.7		

We were interested whether the attitude of PCNPs towards culturally appropriate education contributes to the implementation of CAHE-care in practice. Hence, we compared the attitude of participants who reported the implementation of their intended changes in practice to those PCNPs who did not. The PCNPs who reported the implementation of their CtC statements showed significant attitude changes after three months on three out of 11 questions (p < 0.05) (Table 4, column 6 and 7). Since these questions are associated with objective theme 4 (skills to apply CAHE-care) of this program (Table 1), this result could indicate that a change in attitude is related to a change in intended behavior.

## Discussion

In this study, we describe the development and evaluation of an interactive, comprehensive, and reproducible training program to educate PCNPs in culturally appropriate health care. Although culturally appropriate care is known to improve treatment outcomes of minority patients [16], Dutch Universities of Applied Sciences did not provide regular training on culturally appropriate health education for healthcare providers. The current study is the first to describe and evaluate such a training for PCNPs. We showed that the CAHE-training was well-received by PCNPs and resulted in minor attitude changes towards CAHE. We also found that positive attitude change was correlated with the actual implementation of CAHE in their daily practice. This indicates that the CAHE-training program may prompt attitudinal and behavioral changes with respect to the delivery culturally adapted hypertension care in participants.

In our paper, we provide a detailed description of the background and final design of the intervention. Studies that describe the development and effect of educational interventions generally lack a thorough description of all critical variables involved [28]. The CAHE-training material builds upon our previous obtained knowledge, interrelating culturally adapted care, treatment outcomes, and determinants of treatment outcome [15, 16, 19, 20]. PCNPs were satisfied with the training and specifically appreciated the combination of learning techniques. Participant satisfaction with a provided training program is an important outcome measure in education science. High satisfaction increases the participation level of the participants. However, satisfaction with training does not imply attitude change. With our primary goal of generating an effect on the attitude and behavior of the PCNPs towards CAHE-care in their daily practice, the role-play exercise with a patient-actor was a crucial element of the program. Although participants labeled roleplay as ‘distressing’ beforehand, it was valued highly during the evaluation. Overall, the program was received well by the participants, which aids the acceptation of the content.

The effect of the CAHE-training on the practice of PCNPs was measured using a self-assessment scale, immediately and three months after the program. Self-assessment is the most common approach used to assess cultural competency; it provides a subjective measure and could be subject to bias [29]. The percentage of questionnaires that were returned three months after the training, was low at 36.6%. Based on this sample most attitude scores of the participants did not increase significantly. However, 70% of the PCNPs who returned the CtC questionnaires implemented their CtC-statements. Other studies scoring implementations through CtC statements, report implementation levels of 47% - 87%, which is comparable to the percentage reported here. [30–33] Based on the implementation scores we conclude that the developed training led to changed behavior of the PCNPs in their daily practice. The low response rate may have resulted in an overestimation or underestimation of the effect of attitude and behavior.

The majority (56.8%) of the CtC-statements were linked to theme 4: Skills to apply CAHE-care (Table 5), corresponding to the ‘show how-level’ of the pyramid of Miller [24]. Since the program focused on training by role-play, such a result is not unexpected. However, surprisingly 79.4% of these statements were associated with general MI concepts (not culture specific). Based on constructivist learning theory, we expected that PCNPs could quickly adopt the cultural specific program material since this relates to their pre-existing knowledge and skills in applying MI. The number of statements associated with general MI concepts suggests however that PCNPs were not that competent in MI as expected or the application towards cultural aspects was unclear. In conclusion, the question therefore remains open whether knowledge of MI alone is sufficient to deliver quality care to minority patients.

Comparing PCNPs that reported a behavioral change with those who did not showed that the former care more for patients distrusting the Dutch health care system (Table 4, column 6 and 7). These PCNPs are more considerate of the patients’ cultural background while eliciting patients’ understanding of their illness and their perceptions/concerns regarding prescribed medication. Previously, we showed that similar attitude changes in patients (i.e. patients’ understanding of their illness and concerns and perceptions regarding prescribed medication) are influenced by CAHE-intervention-trained PCNPs [15]. Moreover, these attitude changes positively influence adherence to treatment recommendations and the blood pressure of the patients [14]. We hypothesize that these reoccurring attitude changes are the connection between the CAHE-program of PCNPs and its effect on patients’ outcome.

Here, we showed that the developed training realized desired effects on the behavior of PCNPs. These findings are a valuable addition to our previous work on CAHE. Overall we provide a vast amount of well-documented variables both at the level of health care professionals as well as treatment effects.

## Abbreviations

CAHE-care: delivery of culturally adapted hypertension education to patients in general practice
CAHE-intervention: training in culturally adapted hypertension education piloted in previous intervention study
CAHE-training program: interactive, comprehensive, and reproducible training program on the delivery culturally adapted hypertension education for PCNPs
